# PK-PD Integration Modeling and Cutoff Value of Florfenicol against *Streptococcus suis* in Pigs

**DOI:** 10.3389/fphar.2018.00002

**Published:** 2018-01-17

**Authors:** Zhixin Lei, Qianying Liu, Shuaike Yang, Bing Yang, Haseeb Khaliq, Kun Li, Saeed Ahmed, Abdul Sajid, Bingzhou Zhang, Pin Chen, Yinsheng Qiu, Jiyue Cao, Qigai He

**Affiliations:** ^1^State Key Laboratory of Agricultural Microbiology, College of Veterinary Medicine, Huazhong Agricultural University, Wuhan, China; ^2^National Reference Laboratory of Veterinary Drug Residues and MAO Key Laboratory for Detection of Veterinary Drug Residues, Huazhong Agricultural University, Wuhan, China; ^3^Department of Veterinary Pharmacology, College of Veterinary Medicine, Huazhong Agricultural University, Wuhan, China; ^4^College of Veterinary Medicine, University of lllinois at Urbana – Champaign, Champaign, IL, United States; ^5^College of Veterinary Sciences and Animal Husbandry, Abdul Wali Khan University Mardan, Mardan, Pakistan; ^6^School of Animal Science and Nutritional Engineering, Wuhan Polytechnic University, Wuhan, China

**Keywords:** florfenicol, *Streptococcus suis*, optimal dosages, pharmacokinetic, pharmacodynamic

## Abstract

The aims of the present study were to establish optimal doses and provide an alternate CO_PD_ for florfenicol against *Streptococcus suis* based on pharmacokinetic-pharmacodynamic integration modeling. The recommended dose (30 mg/kg b.w.) were administered in healthy pigs through intramuscular and intravenous routes for pharmacokinetic studies. The main pharmacokinetic parameters of *C*_max_, AUC_0-24h_, AUC, Ke, *t*_1/2ke_, MRT, *T*_max,_ and Cl_b_, were estimated as 4.44 μg/ml, 88.85 μg⋅h/ml, 158.56 μg⋅h/ml, 0.048 h^-1^, 14.46 h, 26.11 h, 4 h and 0.185 L/h⋅kg, respectively. The bioavailability of florfenicol was calculated to be 99.14% after I.M administration. A total of 124 *Streptococcus suis* from most cities of China were isolated to determine the minimum inhibitory concentration (MIC) of florfenicol. The MIC_50_ and MIC_90_ were calculated as 1 and 2 μg/ml. A serotype 2 *Streptococcus suis* (WH-2), with MIC value similar to MIC_90_, was selected as a representative for an *in vitro* and *ex vivo* pharmacodynamics study. The MIC values of WH-2 in TSB and plasma were 2 μg/ml, and the MBC/MIC ratios were 2 in TSB and plasma. The MPC was detected to be 3.2 μg/ml. According to inhibitory sigmoid *E*_max_ model, plasma AUC_0-24h_/MIC values of florfenicol versus *Streptococcus suis* were 37.89, 44.02, and 46.42 h for the bactericidal, bacteriostatic, and elimination activity, respectively. Monte Carlo simulations the optimal doses for bactericidal, bacteriostatic, and elimination effects were calculated as 16.5, 19.17, and 20.14 mg/kg b.w. for 50% target attainment rates (TAR), and 21.55, 25.02, and 26.85 mg/kg b.w. for 90% TAR, respectively. The PK-PD cutoff value (CO_PD_) analyzed from MCS for florfenicol against *Streptococcus suis* was 1 μg/ml which could provide a sensitivity cutoff value. These results contributed an optimized alternative to clinical veterinary medicine and showed that the dose of 25.02 mg/kg florfenicol for 24 h could have a bactericidal action against *Streptococcus suis* after I.M administration. However, it should be validated in clinical practice in the future investigations.

## Introduction

*Streptococcus suis* (*SS*), an important swine industry pathogen could cause significant economic losses worldwide. *SS* is also considered an emerging zoonotic pathogen with the potential to cause disease (e.g., septicemia, meningitis, pneumonia, endocarditis, and arthritis) in humans and pigs (Feng et al., 2014; Goyette-Desjardins et al., 2014). Based on capsular antigens, there are 35 *SS* serotypes, among which serotype 2 is the most virulent, as well as being the dominant pathogenic serotype (Gottschalk et al., 2010, 2013; Feng et al., 2014). Large outbreaks of *SS* serotype 2 have seen in China in 1998 and 2005, resulting in high mortality and morbidity in pigs and humans (Yu et al., 2006; Song et al., 2011). Since *SS* vaccines are either unavailable or unsuitable, antimicrobial agents are used for treating *SS. SS* resistance to antimicrobial agents (macrolides, lincosamides, sulfonamides, and fluoroquinolones) has been reported previously (Varela et al., 2013; Yang et al., 2015). Therefore, it is dire need of the day to establish an optimal dosage regimen for *SS* treatment.

Florfenicol (FF), a structural analog of thiamphenicol, is a broad spectrum antibiotic with strong antibacterial activity not only against gram-negative bacteria such as *Pasteurella multocida*, *Haemophilus sommus*, *Escherichia coli*, but also against gram-positive bacteria such as *SS, Staphylococcus aureus* (Ueda and Suenaga, 1995; Voorspoels et al., 1999). Generally, FF is recommended for treatment of respiratory infections in pigs and cattle (Martel, 1995). Because of its low toxicity and resistance development, its effectiveness is equal to or even better than chloramphenicol, tested in different species (Lobell et al., 1994; Kim et al., 2008, 2011; Birdane and Birdane, 2015). It is recommended for parenteral and I.M inoculation for cure of archentric and respiratory diseases in pigs and cattle with bioavailability greater than 99% (Liu et al., 2003). Previous studies showed that MIC of FF against *Pasteurella multocida*, *Actinobacillus pleuropneumoniae* and *Staphylococcus aureus* were lower than 2 μg/ml (Suzuki et al., 1989; Shin et al., 2005; Nedbalcova and Kucerova, 2013).

The PK profiles of FF had been described in chicken, turkeys, pigs, dogs, rabbits, cattle, and sheep (Liu et al., 2002, 2003; Shen et al., 2002; Anadón et al., 2008; Lane et al., 2008; Park et al., 2008). The published reports revealed that FF had a high concentration, wide and rapid distribution in plasma and peripheral tissue. Moreover, it had shown that FF had a weakly bound to plasma proteins lower than 15% and well-absorbed after I.M and oral administration with a high bioavailability in pigs (Liu et al., 2003; Sidhu et al., 2014). FF could be distributed widely throughout the animals’ bodies, remained high concentration over the MIC of partly respiratory tract pathogens over 24 h (Dorey et al., 2017; Lei et al., 2017). Although some trials have shown the PK of FF in various species including pigs, while the PK data of FF against *SS* is insufficient to accurately predict the effectiveness of this drug.

There are no valuable documented data that relate PK and PD of FF in pigs. PK and PD integration model is considered as an appropriate approach to dose prediction (Dorey et al., 2017; Lei et al., 2017). It is necessary to optimize the dosage calculation procedures to achieve success in clinical therapies and lessen the incidence of antimicrobial agents resistance against the antimicrobial drugs (Burgess, 1999; Yan et al., 2017). The PK-PD model can also avoid the development of resistance and offer optimal dosing strategies (Drusano, 2007; Nielsen and Friberg, 2013). PK-PD analysis has been an effective tool to evaluate the optimal dose for the development of new antimicrobial compounds by the European Medicines Agency (EMA) and Food and Drug Administration (FDA) (Lei et al., 2017; Yan et al., 2017).

It is very common to investigate the antibiotic concentrations in the target site within the animal’s body. Marbofloxacin was studied in ileum content for *Escherichia coli* which was demonstrated that the concentration in ileum content was much higher and more authentic than in plasma in the previously published studies (Wang et al., 2016; Lei et al., 2017; Yan et al., 2017). The PELF of the lungs is also widely considered as the target site of *SS* infection in mammals. However, it is accepted that lung tissues do not act as the bio-phase for pathogens that infect the lungs. Similar to *Pasteurella multocida*, *SS* is a strictly extracellular pathogen (Rodríguez-Ortega et al., 2008; Mandanici et al., 2010; Haas and Grenier, 2015) and mainly localizes in the PELF. Although drug concentrations in the PELF greatly exceed those in the plasma, it could be unable to maintain an effective local extracellular concentration in PELF because of its extremely slow dynamic and release of drug *in vivo. In vivo*, conditions are dynamic and any (slow) release of drug from the lung tissue would not be able to keep an effective drug concentration to local extracellular location (*SS* infection). Kiem and Schentag (2008, 2014) have reported that high PELF drug concentrations are caused by cell lysis during the bronchoalveolar lavage (BAL) procedure to collect PELF, which shows that the measuring procedure for PELF might not be accurate. As the PELF concentration of antibiotics is commonly measured by BAL, technical factors or errors in the method of measurement may create this inaccuracy (Kiem and Schentag, 2008, 2014). Moreover, the difficulty of measuring PELF concentration could also be the most important reason for a proper PK driver selection. The technique might be laborious, intensive, time taking and not applicable for measuring PELF concentration. Evaluation of target tissue is needed. However, there are reaching consensuses, that using plasma concentration of total drug could be used to express PK-PD parameters with microbiological outcomes in animals. Therefore, it is recommended that plasma drug concentrations are more authentic and a suitable final target tissue for PK/PD analysis.

PK/PD cutoff value (CO_PD_) is potentially suitable for the bacterial infection treatment and associated with clinical efficacy. Generally, the cutoff value should be established prior to an antimicrobial drug being clinically used or approved (Jian et al., 2015). The CLSI subcommittee for Veterinary Antimicrobial Susceptibility Testing (VST) develops the cutoff value of veterinary susceptibility, but there are no documented data showing the cutoff value of FF against *SS* in pigs. CO_PD_ is determined by MCS based on PK in target species and PK-PD indices received from the perspective of exposure-response relationship (Müller et al., 2004; Mueller et al., 2004).

There is no previous study investigated the integrated data of PK in pigs with *ex vivo* time course action for FF against the *SS*. The aims of this study were to determine the PK profiles at a single dose of 30 mg/kg by I.M and I.V, and establish optimal dosages for 50 and 90% TAR. It was further aimed to derive a CO_PD_ of FF against *SS* by Monte Carlo simulations by using PK and *ex vivo* PD data.

## Materials and Methods

### Chemicals and Reagents

The standard FF (>97.5%) was purchased from Dr. Ehrenstorfer (Augsburg, Germany). The 30% FF injection was obtained from Wuhan Huisheng Biotechnology, Co., Ltd. All the chemical reagents and organic solvents used were of HPLC grade. To test the liability of this bacterium against FF, each isolate was subculture at least three times in Tryptone Soy Broth (TSB) and Tryptone Soy Agar (TSA; Qingdao Haibo Biological Technology, Co., Ltd.) with 5% calf serum (Zhejiang Tianhang Biotechnology Co., Ltd.).

### Animals

In the present study, a total of eight healthy pigs (four males and four females) were used with an average weight of 15–20 kg and 8–10 weeks of age. These pigs were put in separate houses with the free access to the water and feed without antibiotics. For proper adaptation, these animals were fed with this feed for 1 week before trial. The research was approved by the Ethics Committee of the Faculty of Veterinary Medicine of the Huazhong Agricultural University. All procedures regarding the animal care and testing were carried out according to the recommendation for the care and use of laboratory animals of Hubei provincial public service facilities.

### Bacterial Strain Isolation

A total of 124 *SS* strains were isolated from Chinese pigs (from Anhui, Henan, Hubei, Jiangxi, Sichuan, Hunan, and Guangzhou cities) between 2015 and 2017. Out of these the *SS* WH-2 strain (serotype 2) to study the antimicrobial activity of FF *in vitro*, because its minimal inhibitory concentration (MIC) was similar to its MIC_90_. *E. coli* ATCC 25922 was selected as the reference strain for determination of susceptibility to antibiotics. Polymerase chain reaction (PCR) was used to identify the isolates. Prior to test the MIC, each isolate was subculture three times in TSB and TSA.

### Antimicrobial Susceptibility Monitoring

The susceptibility determination of FF against *SS* was done with the agar dilution method according to the recommendations of the Clinical and Laboratory Standards Institute (CLSI). Strains were injected onto TSA agar plates having calf serum, with twofold serial dilutions of FF (0.0625–32 μg/ml). Those strains with MIC values greater than 32 μg/ml, were re-tested using a broader range of FF dilutions. Inoculated plates were incubated for 48 h at 37°C. The MIC was considered the lowest drug concentrations that caused complete growth inhibition. To confirm the findings of the susceptibility test, *E. coli* (ATCC 25922) was selected as a quality control (QC) strain.

### MIC, MBC, and MPC Determination of WH-2

WH-2 was used for PD study, including MIC, MBC, and MPC determination in TSB and plasma. A 100 μl suspension from 96-well plates of FF, in which the MIC value was measured by the broth dilution technique according to the CLSI guidelines, was diluted 10 times or more with TSB and then spread and counted 10 μl each on the TSA plates for 48 h at 37°C. The minimal bactericidal concentration (MBC) was considered the lowest amount of FF inhibiting bacterial density by 99.9%. The MIC values for WH-2 in broth and serum were also determined using the above process. Then, 10^10^ CFU/ml of *SS* (WH-2) was set to determine the MPC on TSA plates (Blondeau et al., 2010). Furthermore, the suspension was distributed onto TSA, comprising serial dilutions of FF (1 to 32 MIC); MPC was defined as the lowest concentration that inhibits bacterial growth for 96 h at 37°C.

### Bacterial Growth and Killing-Time Curves *in Vitro* and *ex Vivo*

WH-2 was selected to determine the growth-time curve in TSB and plasma. The OD_600 nm_ values were determined for TSB and serum, taking measurements at 0, 2, 4, 6, 8, 10, 12, and 24 h. TSA plates were prepared at various concentrations of FF ranging from 1/4 to 32 MIC. Hundred microliter of the bacterial fluid was diluted using normal sterile saline (10^-1^ to 10^-5^ dilution ratio), then aliquots of the four most diluted samples were applied to the TSA plates at 0, 2, 4, 6, 8, 10, 12, and 24 h of culture, after which the samples were incubated for 48 h at 37°C. In the *ex vivo* time-killing curves, the bacteria (10^6^ CFU/ml) were co-incubated with serum samples received from pigs at different time points (0, 0.25, 0.5, 1, 2, 4, 6, 8, 10, 12, and 24 h) after I.M at a single dose of 30 mg/kg. The *ex vivo* time-killing curve was fitted to a PD model using the hypothesis that a decrease in FF concentration based on incubation time with the inhibitory sigmoid *E*_max_ model.

### Pharmacokinetics Study

#### Experimental Design in Pigs

About eight pigs (four females and four males) weighing 15–20 kg and 4–5 weeks were selected for PK study. Pigs were I.M administrated at a single dose of 30 mg/kg FF. After a radical washout period (2 weeks), the pigs received the same dose of FF with I.V administration. Blood samples (5 ml) were mildly collected from a jugular vein into the anticoagulant tube. Blood samples (5 ml) were obtained at 0.25, 0.5, 1, 2, 4, 6, 8, 10, 12, 24, 36, 48, 72, 96, 120, 144, and 196 h after I.M and I.V administration.

#### Blood Treatment

The collected blood samples with the anticoagulant were centrifuged at 3000 rpm for 10 min to obtain the blood plasma. Then, 2 ml of dichloromethane was added to 0.5 ml of plasma, the tubes were vortexed for 2 min and centrifuged for 10 min at 5000 rpm. This method was repeated two times. The dichloromethane phase was shifted to a sterile tube and evaporated under nitrogen in a thermostat water bath at 60°C. A portion of the mobile phase (0.5 ml) was then added to the dried tube to dissolve the sample. The resulting samples were filtered through membrane filters having a pore size of 0.22 μm and analyzed by HPLC.

#### FF Binding to Serum Protein

Serum protein binding of FF was determined in triplicate on each of nine pooled blood samples, harvested at predetermined times from the eight pigs used in the PK study. For each sample, the total concentration of FF was determined as follows. Samples were centrifuged at 4000 *g* for 10 min using an Amicon Ultra Centrifugal Filter (Ultracel 10K; Millipore Limited, Watford, Hertfordshire, United Kingdom) and FF concentration re-determined on the ultra-filtrate.

#### HPLC Conditions for FF and Pharmacokinetic Analysis

A C18 reverse phase column (250 mm × 4.6 mm, i.d., 5 μm, Agilent, United States) was used for HPLC, which was executed at a detection wavelength of 233 nm at 30°C. The mobile phase comprised of acetonitrile (phase A) and 0.1% formic acid (phase B) (v:v, 67:33) with a mobile phase flow rate of 1 ml/min. The HPLC method authentications of FF in plasma were determined by the standard external method. The linear range for the standard curve of FF ranged from 0.05 to 10 μg/ml in serum was detected by HPLC, and the linear regression, curve recovery and coefficient of variation were calculated. The recovery ratios were the specific values of calculated peak area in serum to standard with different drug concentrations (0.05, 0.1, 0.5, 1, 5, and 10 μg/ml). The LLOD was the lower detected concentration at the value of the signal to noise ratio (S/N) > 3 in serum. The LLOQ was the lower detected concentration at the value of S/N > 10 in serum.

The parameters of PK were calculated from the plasma FF concentrations using WinNonlin software (version 5.2.1, Pharsight Corporation, Mountain View, CA, United States). To select the appropriate PK models, the drug concentrations were recorded in the semi-logarithmic graphs. PK parameters obtained by the least squares regression analysis were calculated using WinNonlin software.

### PK/PD Integration Analysis

For antibiotic, the pharmacokinetic or pharmacodynamic parameters of which dependents on the concentration, the PK/PD index should be AUC_0-24h_/MIC and *C*_max_/MIC (Andraud et al., 2011; Yohannes et al., 2015). The AUC_0-24h_/MIC and *C*_max_/MIC were used as the combined PK/PD parameters, which were measured for each dose of the time-killing curve. Using WinNonlin software, an inhibitory sigmoid *E*_max_ model was selected to determine integration of AUC_0-24h_/MIC ratio *in vitro* and bacteria count change (CFU/ml) in the ileum contents during 24 h incubation period (Aliabadi and Lees, 2001, 2002; Aliabadi et al., 2003). The appropriate model equation was designated as (Equation 1):

$$E=E_{\max}=\left(1-\frac{C^{N}}{C^{N}+{EC}_{50}^{N}}\right)$$

where *E* represents the effect of the antimicrobial agent calculated as the log_10_ difference in bacterial number before and after a 24 h incubation time *in vitro*. The EC_50_ value indicates that the AUC_0-24h_/MIC value reached 50% of the *E*_max_, *C* represents the AUC_0-24h_/MIC ratio, and *N* shows Hill coefficient.

### Dose Estimations

To deduce an optimal regimen, the following formula was applied to evaluate dosages in different magnitudes of efficiency (*E* = 0, no change in the bacterial count; *E* = -1, 99.9% reduction in the count; *E* = -3, 99.99% reduction):

$$Dose=\frac{(AUC/MIC)\cdot {MIC}_{90}\cdot CL}{fu\cdot F}$$

where AUC/MIC shows the targeted end-point for optimal efficacy, MIC represents the minimum inhibitory concentration, CL displays clearance per day, *fu* shows the free fraction of the drug in plasma, and F signifies the bioavailability.

The probabilities of distribution of predicted daily dosages were conducted for 100000 trails to achieve 50 and 90% TAR for bacteriostatic, bactericidal and bacterial elimination effects by using Monte Carlo Simulations in Oracle Ball (Oracle Corporation, Redwood Shores, CA, United States).

### Monte Carlo Analysis and Determination of Pharmacodynamics Cutoff Value

A Monte Carlo simulation (MCS) with 10,000 iterations was conducted using Crystal Ball software (version 7.2.2) (Oracle, United States) based on PK parameters and calculated PK/PD targets (AUC_24h_/MIC) when it appeared bactericidal action (*E* = -3) (Andes et al., 2004a,b; Zhang P. et al., 2016). The AUC_24h_ was assumed to be log-normally distributed for the mean values and confidence intervals (CI). The CO_PD_ was defined as the MIC at which the PTA reached up to 90%, according to the CLSI guidelines described in previous reports by Turnidge and Paterson (2007) and Zhang P. et al. (2016).

### Statistical Analysis

MIC_90_ was measured using SPSS software by calculation of cumulative frequencies, and the statistical analyses were done by the Student’s *t*-test and Bonferroni using Prism software (Graphpad Software Inc., London, United Kingdom). *P*-values < 0.05 were considered to indicate statistically significant differences.

## Results

### MICs Distribution of FF against *SS*

**FIGURE 1:**
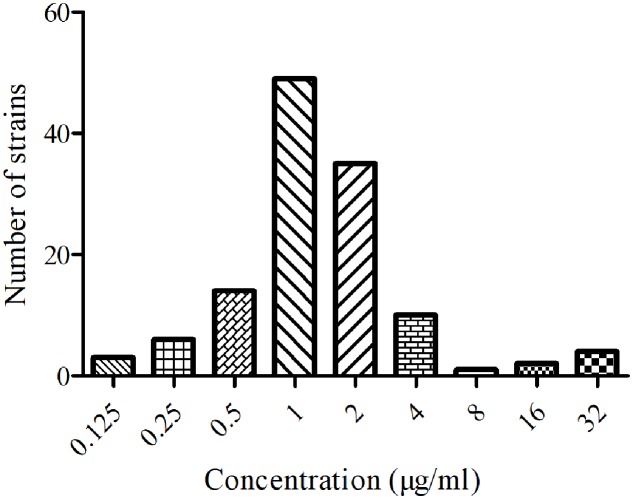
Minimum inhibitory concentration distribution of FF against *SS* (*n* = 124).

The MICs of 124 isolated *SS* were ranged from 0.125 to 32 μg/ml. The MIC_50_ and MIC_90_ values were calculated to be 1 and 2 μg/ml. The distribution of FF against *SS* was shown in **Figure 1**. The main serotypes of these isolated SS were determined to be 2 and 4 types.

### MIC, MBC, and MPC of WH-2 *in Vitro* and *ex Vivo*

The MIC and MBC of FF against WH-2 were both 2 and 4 μg/ml in TSB and plasma. In addition, the MPC of FF against WH-2 was 3.2 μg/ml.

### Bacterial Growth and Killing-Time Curves *in Vitro* and *ex Vivo*

**FIGURE 2:**
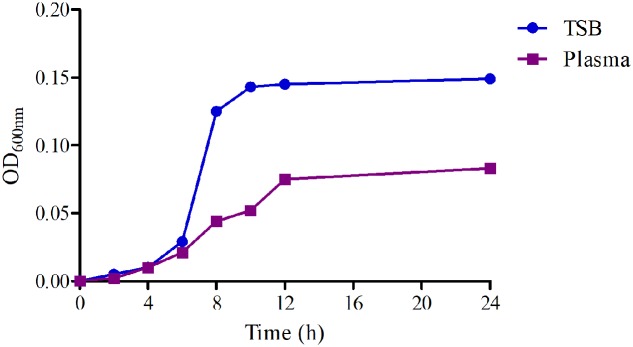
The growth-time curves of WH-2 in TSB (*in vitro*) and plasma (*in vivo*).

The growth-time curves in TSB and plasma were shown in **Figure 2**. Obviously, the logarithmic phases of WH-2 in TSB and serum were from 2 to 10 h and 2 to 12 h, respectively. The total bacterial amount and growth rate were higher in TSB than in serum. The findings suggest that serum might inhibit bacterial growth.

**FIGURE 3:**
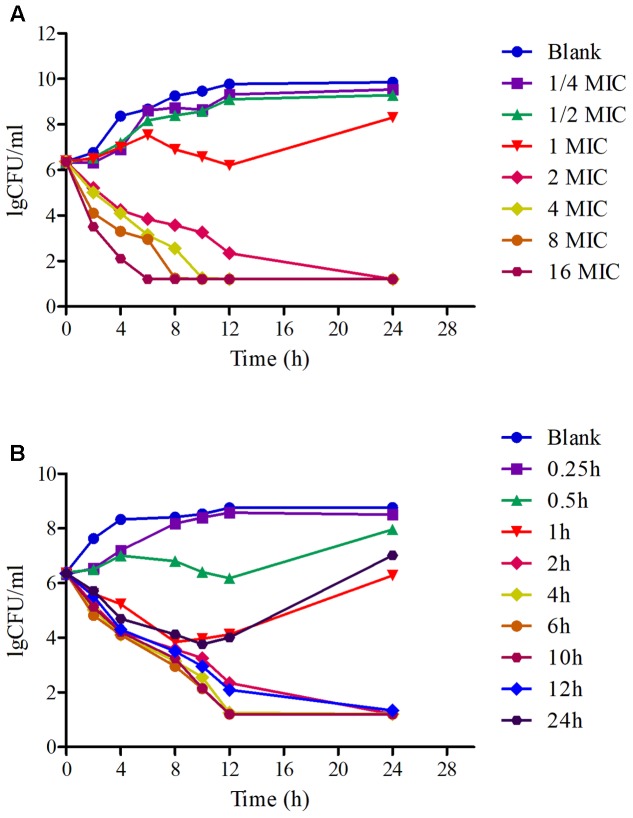
The killing-time curves of FF against WH-2 in TSB and plasma. **(A)** Represented the curve in TSB, **(B)** represented the curve in plasma.

The *in vitro* and *ex vivo* killing-time curves of FF against WH-2 were illustrated in **Figure 3**. The concentration-dependent bactericidal activity was observed after the *in vitro* profiles, since the increase in drug concentrations produced more rapid and radical disruptive effects. Obviously, when the concentrations were over or equal to 2 MIC, the inhibition of bacterial growth was significantly declined to the undetectable level (<10 CFU/ml) after 24 h in **Figure 3A**. The *ex vivo* killing-time curve of FF against WH-2 in plasma was shown in **Figure 3B**. The bacteria were reduced drastically to the undetectable limit (<10 CFU/ml) after exposure to the plasma collected between 2 and 12 h after I.M administration. These suggested that FF had an activity of killing that depends on the concentration in the *ex vivo* environment, consistent with *in vitro* bactericidal activity.

### PK Analysis of FF in Plasma after I.M and I.V Administrations

**FIGURE 4:**
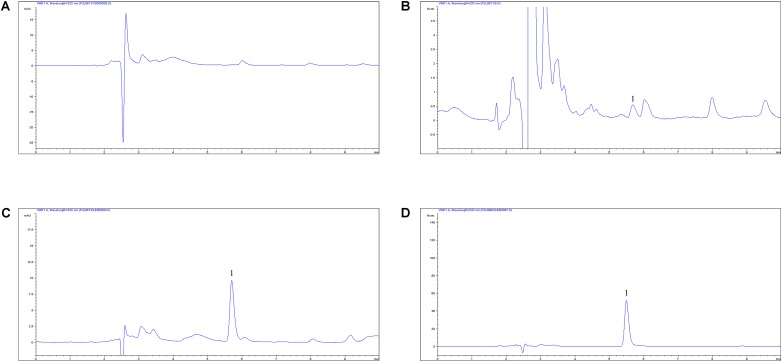
The HPLC method for FF quantification in plasma. **(A)** Blank sample, **(B)** plasma sample at the LLOQ of 0.05 μg/ml, **(C)** plasma sample at the 2 h after I.M administration, **(D)** plasma sample at the 2 h after I.V administration, 1, the retention time of 5.5 min.

The proposed HPLC methods for the detection of FF were suitable in the plasma. These methods have demonstrated specificity and high rates of recovery (>90%) in plasma in accordance with the residue guidelines of the Veterinary Pharmacopoeia of the Department of Agriculture and the Pharmacopoeia of the United States (Gad, 2014; Lei et al., 2017 Evaluation of OTC). Moreover, these also showed a decent linear association from 0.05 to 10 μg/ml in the plasma. The chromatograms of the quantification methods were shown in **Figure 4** and it represented blank in **Figure 4A**, the lower limit of quantification (LLOQ) in **Figure 4B**, measured sample after I.M administration in **Figure 4C** and measured sample after I.V administration in **Figure 4D**. The lower limit of determination (LLOD) and LLOQ in plasma were 0.25 and 0.05 μg/ml and the retention time of FF detection was 5.5 min. In addition, the typical regression equation was *y* = 23.43*x* + 0.685, *R*^2^ = 0.9998 in serum.

**FIGURE 5:**
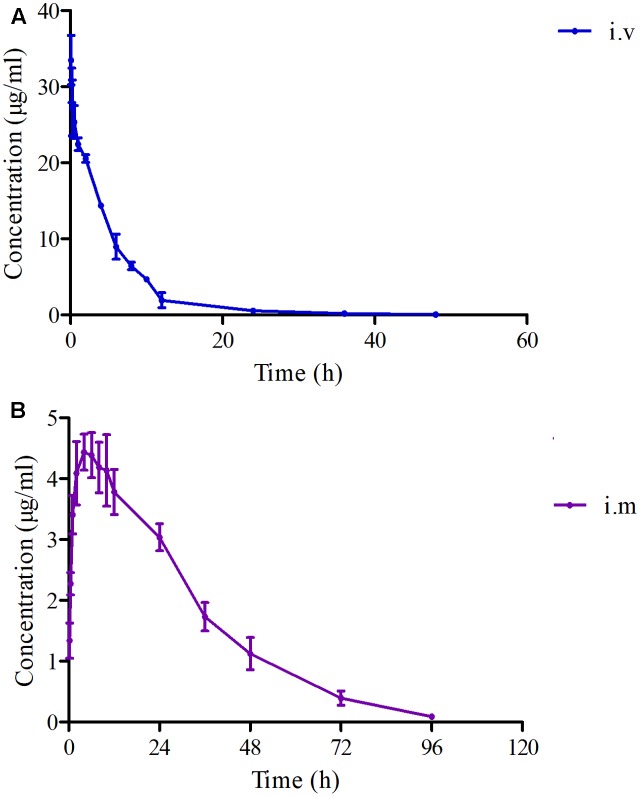
The curves of FF concentrations-time in plasma after I.V and I.M administrations (30 mg/kg) at 0, 0.25, 0.5, 1, 2, 4, 6, 8, 10, 12, 24, 36, 48, 72, and 96 h (*n* = 8). **(A)** The curve after I.V administration, **(B)** the curve after I.M administration.

In the pooled serum samples of eight pigs study, the percentage of free drug concentration was 85 ± 3.3%. The principal PK parameters were exhibited in **Table 1** with using non-compartment models analysis after I.V and I.M administrations. The main pharmacokinetic parameters of *C*_max_, AUC_0-24h_, AUC, Ke, *t*_1/2ke_, MRT, *T*_max_, and Cl_b_ were 4.44 μg/ml, 88.85 μg⋅h/ml, 158.56 μg⋅h/ml, 0.048 h^-1^, 14.46 h, 26.11 h, 4 h, and 0.185 L/h⋅kg, respectively and the bioavailability of FF was calculated to be 99.14% after I.M administration (**Table 1**). Moreover, the main pharmacokinetic parameters of AUC_0-24h_, AUC, Ke, *t*_1/2ke_, MRT, and Cl_b_ were 154.18 μg⋅h/ml, 159.93 μg⋅h/ml, 0.106 h^-1^, 6.54 h, 5.85 h, and 0.187 L/h⋅kg, respectively, after I.V administration (**Table 1**). The mean ± SD of FF concentration-time profiles were shown in **Figure 5** after I.M and I.V administration.

**Table 1:** The PK parameters of FF after I.M and I.V administrations (30 mg/kg) in pigs (*n* = 8).

Parameters	Unites	I.M	I.V
Ke	h^-1^	0.048 ± 0.004	0.106 ± 0.015


*t*_1/2ke_	H	14.46 ± 1.84	6.54 ± 0.75


Cl_b_	L/h⋅kg	0.185 ± 0.027	0.187 ± 0.032


AUC	μg⋅h/ml	158.56 ± 13.46	159.93 ± 13.28


AUC_0-24h_	μg⋅h/ml	88.85 ± 6.72	154.18 ± 11.12


*C*_max_	μg/ml	4.44 ± 1.02	–


*T*_max_	H	4 ± 1.34	


MRT	H	26.11 ± 3.24	5.85 ± 1.17


*F*	–	99.14%	–




*Ke, presented elimination rate constant; *t*_*1/2ke*_, presented elimination half-life; Cl_*b*_, presented body clearance; AUC, presented area under the curve; AUC_0-*24h*_, presented area under the curve from 0 to 24 h; *C*_*max*_, peak concentration; *T*_*max*_, the time of maximum serum concentration; the MRT, presented mean residence time; F, the bioavailability.*

### PK-PD Integration Modeling

**FIGURE 6:**
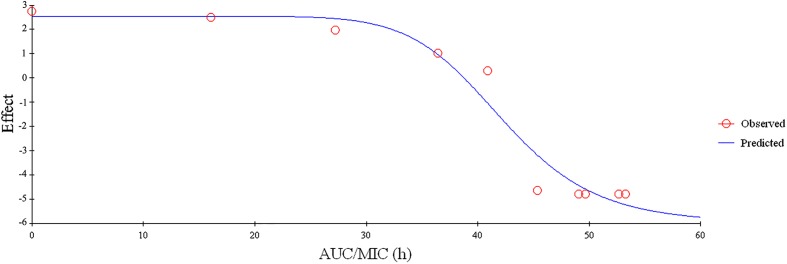
Plots of *ex vivo* AUC/MIC ratios versus the amount difference of FF against *SS* within 24 (*n* = 8).

Since FF showed a concentration-dependent, the PK-PD parameters of AUC_0-24h_/MIC, AUC_0-24h_ /MPC or *C*_max_/MIC, *C*_max_ /MPC would be more appropriate for the PK-PD modeling. In addition, the values of AUC_0-24h_/MIC, AUC_0-24h_ /MPC and *C*_max_/MIC, *C*_max_/MPC received from PK in plasma and *ex vivo* PD were calculated to be 2.34, 1.41, 44.43, and 27.77 h, respectively (**Table 2**). The association between antimicrobial efficiency and the *ex vivo* PK-PD parameters of AUC_0-24h_/MIC ratios were simulated by using the model of *E*_max_ inhibitory sigmoid. The parameters of the Hill coefficient *N*, *E*_0_, *E*_max_ and AUC_0-24h_/MIC values of the model for three levels of the growth inhibition were shown in **Figure 6** and **Table 3**. The standards of the AUC_0-24h_/MIC ratio were calculated for the bacteriostatic activity (*E* = 0), bactericidal activity (*E* = -3), and bacterial elimination (*E* = -4) were 37.89, 44.02, and 46.42 h in **Table 3**.

**Table 2:** The main PK/PD integration parameters for FF against *SS* in plasma after I.M administration (30 mg/kg) in pigs (*n* = 8).

Parameters	Unites	Mean ± *SD*
*C*_max_/MIC	–	2.34 ± 0.63
*C*_max_/MPC	–	1.41 ± 0.24
AUC_0-24h_/MIC	H	44.43 ± 5.15
AUC_0-24h_/MPC	H	27.77 ± 3.45


**Table 3:** The main parameters of PK/PD modeling for FF against *SS ex vivo* after I.M administration in pigs (*n* = 8).

Parameters	Unites	Mean ± *SD*
*E*_max_	Lg CFU/ml	2.36 ± 0.48


EC_50_	h	42.22 ± 1.20


*N*	–	9.89 ± 1.15


AUC_0-24h_/MIC for bacteriostatic (*E* = 0)	h	37.89 ± 4.25


AUC_0-24h_/MIC for bactericidal (*E* = -3)	h	44.02 ± 4.85


AUC_0-24h_/MIC for eradication (*E* = -4)	h	46.42 ± 6.45




**E*_*max*_, presented the Lg change in bacterial counts of blank sample; EC_*50*_, presented the value to achieve 50% maximal antibacterial effect; *N*, presented the Hill coefficient, Hill, the dose-response curve slop.*

### Doses Estimation

**FIGURE 7:**
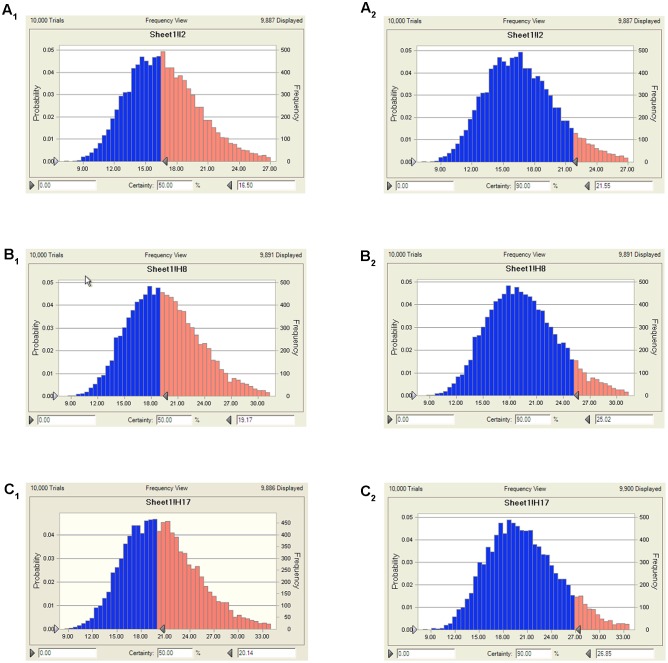
The predicted doses of FF curing SS for 50 and 90% TAR. **(A_1_)** Presented the predicted population dose for bacteriostatic at 50% target, **(A_2_)** presented the predicted population dose for bacteriostatic at 90% target, **(B_1_)** presented the predicted population dose for bactericidal at 50% target, **(B_2_)** presented the predicted population dose for bactericidal at 90% target, **(C_1_)** presented the predicted population dose for elimination at 50% target, **(C_2_)** presented the predicted population dose for elimination at 90% target.

According to AUC_0-24h_/MIC ratios for these three levels of antibacterial activity, measured from the modeling of PK-PD integration and the *ex vivo* PD distribution data by using Monte Carlo Simulations in Oracle Crystal Ball, the predicted daily doses were shown in **Table 4**. The distribution of predicted population dose (AUC_0-24h_/MIC) values of FF curing *SS* for 50 and 90% target were calculated and observed in **Table 4** and **Figure 7**. Based on dose equations and Monte Carlo Simulations, we predicted the FF dosages needed for bactericidal, bacteriostatic, and elimination activity against *SS* WH-2 as 16.5, 19.17, and 20.14 mg/kg b.w. for the 50% target, and 21.55, 25.02, and 26.85 mg/kg b.w. for the 90% target, respectively (**Table 4**).

**Table 4:** The predicted daily doses of FF curing *SS.*

Predicted doses (mg/kg b.w.)	Target ratios	
	
	50%	90%
Bacteriostatic (*E* = 0)	16.50	21.55


Bactericidal (*E* = -3)	19.17	25.02


Eradication (*E* = -4)	20.14	26.85




### CO_PD_ Determination with MCS

The cumulative target achievement calculated from *ex vivo* PD and PK data determined in plasma was 44.02 h by using Crystal Ball software (**Table 4**). The PTA was calculated as 52.25% when the MIC at the value of 2 μg/ml, but 100% of the value of 1 μg/ml. Consequently, a PTA ≥ 90% could be obtained for isolates with MIC ≤ 1 μg/ml in pig serum after I.M. administration at a dose of 30 mg/kg b.w. (**Table 5**). Therefore, the CO_PD_ of FF against *SS* could be calculated as 1 μg/ml.

**Table 5:** The AUC_24_/MIC values calculated with Monte Carlo simulation for PTA.

Doses	Effect	MIC (μg/ml)				
		
		0.25	0.5	1^∗^	2	4
30 mg	Eradication	100%	100%	100%	53.25%	0%


*^∗^Represent the value of CO_*PD*_ breakpoint.*

## Discussion

To date, serotype 2 *SS* is the most contagious and the prevailing pathogenic serotype which is related with various clinical infections (septicemia, meningitis, pneumonia, endocarditis, and arthritis) both in human and animals. In addition, the higher levels of resistance of *SS* to antimicrobials such as macrolides, lincosamides, and sulfonamides including FF, has been reported and limited to another choice for treatment (Mueller et al., 2004). Furthermore, the control of *SS* is difficult due to its high resistance and rapid spread to common antimicrobial drugs resulting from overuse and misuse (Burgess, 1999). In this study, the MIC_50_ and MIC_90_ of tested isolated strains were 1 and 2 μg/ml (**Figure 1**), respectively. It suggests that FF possessed a satisfactory potency against these *SS* isolates. However, according to the EUCAST clinical cutoff values guidance document (EMA, clinical breakpoint) of chloramphenicol against *SS* (resistance, MIC > 8 μg/ml) (EUCAST, 2014), there were resistant isolates found in this study (**Figure 1**). Therefore, as a classical broad-spectrum antibiotic for FF, it is essential to conduct an updated optimal dosage to effectively manage resistance development.

In this study, MIC_50_ and MIC_90_ were calculated based on the MIC distribution of *SS* from most cities of China, and the WH-2 a serotype 2 whose MIC value was similar to MIC_90_ with a high pathogenicity conducted in mice (data not shown) was selected for *in vitro* and *ex vivo* PD study. In previously published reports, the pathogen was selected from 6 to 10 isolates of target animals or simply a standard strain (Yohannes et al., 2015; Hossain et al., 2017; Zhou et al., 2017). However, the WH-2 strain used in the study could be considered as a more favorable representation strain for PD with high virulent and tested MIC similar to MIC_90_. Compared the growth activity of WH-2 *in vitro* to *ex vivo*, it could be observed that the total bacteria counts for HB2 were lower in serum than in TSB, whereas the MIC values were similar (**Figure 2**). This might be due to serum effect potency, as was reported by Toutain et al. (2017). It was observed that there was no any susceptibility cutoff value of FF against *SS* according to the CLSI and EUCST guidance documents. The CO_PD_ of FF against was calculated to be 0.5 μg/ml with PK *in vivo* and *ex vivo* PD data and the value was lower than the similar drug “chloramphenicol” (MIC ≤ 4 μg/ml) reported by EUCAST and CLSI M100-S23 guidance documents. This result of CO_PD_ value (0.5 μg/ml) for FF could provide an alternative susceptibility cutoff value for isolated strains in clinical veterinary medicine.

As it is known, that FF is a wide spectrum, synthetic antibacterial. It is usually used in bovine and pigs by intramuscular route administration and in fish by administration via drinking water (EMA, 1999). In this study, FF was I.M and I.V administrated with a single dosage of 30 mg/kg, the PK data in plasma for *C*_max_, AUC_0-24h_, AUC, Ke, *t*_1/2ke_, MRT, *T*_max_, and Cl_b_ were 4.44 μg/ml, 88.85 μg⋅h/ml, 158.56 μg⋅h/ml, 0.048 h^-1^, 14.46 h, 26.11 h, 4 h, and 0.185 L/h⋅kg by I.M administration, and 154.18 μg⋅h/ml, 159.93 μg⋅h/ml, 0.106 h^-1^, 6.54 h, 5.85 h, and 0.187 L/h⋅kg by I.V administration (**Table 1**). In the present study the F% (99.14%) was found almost similar to the reports (103.74%) documented by Liu et al. (2003) in pigs administered through I.M route, but higher than that in chickens (87%) in the previous report (Shen et al., 2002). The difference may be caused by the animal species. Moreover, the *t*_1/2ke_ (14.46 h) was similar to those (14.27, 16.84, and 16.49 h) in the previously described reports (Liu et al., 2003; Yang et al., 2009; Sidhu et al., 2014). However, the Cl_b_ (0.185 L/h⋅kg) in this study was lower than those (0.3, 0.22, and 0.23 L/h⋅kg) in pigs (Liu et al., 2003; Zhang Q. et al., 2016; Dorey et al., 2017), and that (0.49 L/h⋅kg) in beagle dogs (Kim et al., 2011), (0.23 L/h⋅kg) in calves (Sidhu et al., 2014), in the previously published reports. Furthermore, the AUC (158.56 μg⋅h/ml) and *T*_max_ (4 h) in this study was near three to four times than those (68.61 μg⋅h/ml, 0.91 h) while the dosage (30 mg/kg) was 1.5 time than that (20 mg/kg) reported by Liu et al. (2003). These result revealed that this kind of FF may have sustained release with a lower body clearance, higher AUC and longer *T*_max_. The plasma drug concentration after 24 h (near 3 μg/ml) was much higher than the MIC_90_ of the *SS* strains in **Figure 1**. These indicated that FF could have strong inhibitory effect *in vivo* after 30 mg/kg I.M administration and remain high plasma drug concentrations.

PK-PD integration modeling can be used to select rational dose regimes in veterinary medicine (Toutain et al., 2002) and the drug concentration detection at the infection site is preferred for PK-PD modeling (Lei et al., 2017). However, for respiratory tract infection, especially caused by extracellular bacteria (e.g., *SS*, *Haemophilus parasuis*, and *Pasteurella multocida*), the target tissue (PELF) is unsuitable for PK-PD because of slow drug release and a dynamic *in vivo* environment. *In vivo* conditions are dynamic and any (slow) release of drug from the lung tissue would not be able to keep an effective drug concentration to local extracellular location (*SS* infection). Kiem and Schentag have reported that high PELF drug concentrations are caused by cell lysis during the bronchoalveolar lavage (BAL) procedure to collect PELF (Kiem and Schentag, 2008); it means that the measuring procedure for PELF concentration might be not accurate. As the PELF concentration of antibiotics is commonly measured by BAL, technical factors or errors in the method of measurement may create this inaccuracy. Therefore, PELF is unable to maintain effective local extracellular concentration, although higher drug concentrations have been reported for PELF than serum (Nowakowski et al., 2004; Villarino et al., 2013a,b; Toutain et al., 2016). In addition, it has been reported that FF has a high bioavailability (nearly 90–112%) after oral or intramuscular injection. So FF bioavailability was estimated as 99.14% after an I.M administration of 30 mg/kg dose. Thus, the serum could be suggested as an optimal site for PK and PD studies of FF. Few reports on PK-PD modeling have been established for FF against *Actinobacillus pleuropneumoniae* and *Pasteurella multocida* in pigs (Dorey et al., 2017). However, there were no PK-PD integration modeling analyses for FF against *SS*.

The PK-PD of antibiotics differed from other drugs in that, that the target species were different from its host. Moreover, these thresholds (PK-PD target) could be different for different antibiotics. As a matter of fact, PK-PD integration may be considered as a complementary approach to PK-PD modeling for predicting the adequacy of a dosage regimen in clinical subjects (Lees et al., 2015; Dorey et al., 2017). Since there were differences in the immune status of target animals and pathogens, therefore the PK-PD index values for different antibiotic against specific pathogen were different. Therefore, it was significant to study the PK-PD index for FF against *SS*. For the PK-PD integration process, the PK parameters of free FF in plasma were integrated with the *ex vivo* MIC with WH-2 which was regarded as a typical pathogenic strain of *SS*. In this study, the ratios of *C*_max_/MIC, *C*_max_/MPC, AUC_0-24h_/MIC and AUC_0-24h_/MIC were calculated as 2.34, 1.41, 44.43, and 27.77 h, respectively (**Table 2**). These results revealed that the *C*_max_ could be over 1–2 times higher to the MIC_90_ and MPC of the population of *SS* and the FF concentrations could be kept over MIC_90_ or MPC for over 24 h. It indicated that FF had a strong antibacterial effect on *SS*. In terms of the antibacterial properties of FF against *SS*, the inhibitory sigmoid *E*_max_ model was used to model the PK-PD. The inhibitory sigmoid *E*_max_ model showed a favorable correlation (*R*^2^ = 0.999) between the observed and predicted antibacterial efficacy of FF against *SS* (**Figure 6**). This indicated that the AUC_0-24h_/MIC could be the optimal PK-PD index for PK-PD integration model. The AUC_0-24h_/MIC received *in vivo* PK and *ex vivo* PD study of FF requiring bactericidal and eradication actions to *SS* were 44.02 and 46.42 h, respectively. It was observed that the target for bactericidal and eradication actions of FF against *SS* were similar or higher than the AUC_0-24h_/MIC (44.43 h) *in vivo* after I.M administrated 30 mg/kg. These results suggest that the dosage used in this study (30 mg/kg) could cover and guarantee clinical efficacy against infections associated with WH2 whose MIC was 2 μg/ml. According to the dosage calculation equation and the Monte Carlo simulations, the predicted daily dosage for 50 and 90% TAR to provide a 3 log^10^ reduction (*E* = -3) in bacterial amounts were calculated to be 19.17 and 25.02 mg/kg, respectively (**Table 4**). However, an attention should be paid that the bacterial endpoint *in vivo* might differ from the predicted doses calculated from *ex vivo* data. It was further observed that the animals’ immune system may play an important role in bacterial eradication (Garrison et al., 2002; Nielsen and Friberg, 2013; Lei et al., 2017; Yan et al., 2017). In addition, the calculated PK and PD data from small sample size could not support the conclusions in this study. Therefore, these predicted daily dosages should also be verified in clinical practice.

## Conclusion

It has been demonstrated that the misuse of antibiotics increases the antimicrobial resistance (Nguyen et al., 2012; Zhang et al., 2013). The findings of the present study demonstrate that a single I.M dosage of FF (30 mg/kg) could cover and guarantee bactericidal effect against *SS*. However, in order to reduce the resistance development and risk, a lower and precise dosage of 25.02 mg/kg may be lower toxicity and more effective to administer against *SS* via the intramuscular route. Furthermore, the CO_PD_ of FF against *SS* could be calculated as 1 μg/ml which could be considered as a preliminary susceptibility cutoff value. These calculated doses estimates should also be validated in clinical practice in the further study.

## Author Contributions

QL, SA, YQ, and JC conceived this work. ZL and JC designed the experiment. BY, SY, KL, BZ, and ZL performed the experiments. ZL wrote the manuscript. QH, HK, AS, PC, and JC improved the language. All authors reviewed the manuscript.

## Conflict of Interest Statement

The authors declare that the research was conducted in the absence of any commercial or financial relationships that could be construed as a potential conflict of interest.

## Abbreviations

AUC: area under the curve
AUC_24h_: area under the curve at 24 h
BA or F: bioavailability
BAL: bronchoalveolar lavage
b.w.: body weight
CFU: colony forming unit
Cl_b_: relative total systemic clearance
*C*_max_: peak concentration
CO_PD_: PK/PD cutoff
FF: florfenicol
HPLC: high-performance liquid chromatography
I.M: intramuscular
I.V: intravenous
Ke: elimination rate constant
MIC: minimum inhibitory concentration
MPC: mutant prevention concentration
MRT: mean residence time
PD: pharmacodynamic
PELF: pulmonary epithelial lining fluid
PK: pharmacokinetic
PK/PD: pharmacokinetics-pharmacodynamics
PTA: probability of target attainment
*SS*: *Streptococcus suis*
*t*_1/2ke_: terminal half-life
TAR: target attainment rates
*T*_max_: time to peak concentration
TSA: Tryptic Soy Agar
TSB: Tryptic Soy Broth
